# Hepatic safety and tolerability of cipargamin (KAE609), in adult patients with *Plasmodium falciparum* malaria: a randomized, phase II, controlled, dose-escalation trial in sub-Saharan Africa

**DOI:** 10.1186/s12936-021-04009-1

**Published:** 2021-12-20

**Authors:** Gilles Ndayisaba, Adoke Yeka, Kwaku Poku Asante, Martin P. Grobusch, Etienne Karita, Henry Mugerwa, Stephen Asiimwe, Abraham Oduro, Bakary Fofana, Seydou Doumbia, Jay Prakash Jain, Sarita Barsainya, Gerd A. Kullak-Ublick, Guoqin Su, Esther K. Schmitt, Katalin Csermak, Preetam Gandhi, David Hughes

**Affiliations:** 1 Rinda Ubuzima, Kigali, Rwanda; 2 Infectious Diseases Research Collaboration, Masafu, Uganda; 3 Kintampo Health Research Centre, Kintampo North Municipality, Kintampo, Ghana; 4 Centre de Recherches Médicales en Lambaréné, Lambaréné, Gabon; 5 Amsterdam University Medical Centers, Amsterdam, The Netherlands; 6 University of Tübingen, Tübingen, Germany; 7 Center for Family Health Research, Kigali, Rwanda; 8 Joint Clinical Research Centre, Kampala, Uganda; 9 Kabwohe Clinical Research Centre and Mbarara University of Science and Technology, Mbarara, Uganda; 10 Navrongo Health Research Centre, Navrongo, Ghana; 11 Malaria Research and Training Centre, Sotuba, Mali; 12 University Clinical Research Centre, Bamako, Mali; 13 Novartis Institutes for BioMedical Research, Emeryville, CA USA; 14 Novartis Healthcare Pvt Ltd, Hyderabad, India; 15 Novartis Pharma AG, Novartis Campus, 4056 Basel, Switzerland; 16 University Hospital Zürich and University of Zürich, Zürich, Switzerland; 17 Novartis Pharmaceuticals Corporation, East Hanover, NJ USA; 18 Sandoz AG, Basel, Switzerland

**Keywords:** Cipargamin, Malaria, KAE609, Hepatic safety, Phase II randomized controlled trial, *Plasmodium falciparum*

## Abstract

**Background:**

The novel anti-malarial cipargamin (KAE609) has potent, rapid activity against *Plasmodium falciparum*. Transient asymptomatic liver function test elevations were previously observed in cipargamin-treated subjects in two trials: one in malaria patients in Asia and one in volunteers with experimentally induced malaria. In this study, the hepatic safety of cipargamin given as single doses of 10 to 150 mg and 10 to 50 mg once daily for 3 days was assessed. Efficacy results, frequency of treatment-emerging mutations in the *atp4* gene and pharmacokinetics have been published elsewhere. Further, the R561H mutation in the *k13* gene, which confers artemisinin-resistance, was associated with delayed parasite clearance following treatment with artemether–lumefantrine in Rwanda in this study. This was also the first study with cipargamin to be conducted in patients in sub-Saharan Africa.

**Methods:**

This was a Phase II, multicentre, randomized, open-label, dose-escalation trial in adults with uncomplicated falciparum malaria in five sub-Saharan countries, using artemether–lumefantrine as control. The primary endpoint was ≥ 2 Common Terminology Criteria for Adverse Events (CTCAE) Grade increase from baseline in alanine aminotransferase (ALT) or aspartate transaminase (AST) during the 4-week trial.

**Results:**

Overall, 2/135 patients treated with cipargamin had ≥ 2 CTCAE Grade increases from baseline in ALT or AST compared to 2/51 artemether–lumefantrine patients, with no significant difference between any cipargamin treatment group and the control group. Cipargamin exposure was comparable to or higher than those in previous studies. Hepatic adverse events and general safety and tolerability were similar for all cipargamin doses and artemether–lumefantrine. Cipargamin was well tolerated with no safety concerns.

**Conclusions:**

This active-controlled, dose escalation study was a detailed assessment of the hepatic safety of cipargamin, across a wide range of doses, in patients with uncomplicated falciparum malaria. Comparison with previous cipargamin trials requires caution as no clear conclusion can be drawn as to whether hepatic safety and potential immunity to malaria would differ with ethnicity, patient age and or geography. Previous concerns regarding hepatic safety may have been confounded by factors including malaria itself, whether natural or experimental infection, and should not limit the further development of cipargamin.

*Trial registration *ClinicalTrials.gov number: NCT03334747 (7 Nov 2017), other study ID CKAE609A2202

**Supplementary Information:**

The online version contains supplementary material available at 10.1186/s12936-021-04009-1.

## Background

There is a need to develop new anti-malarials as emerging resistance to artemisinin derivatives threatens to undermine progress in malaria control. Cipargamin (KAE609/NITD609) is a novel anti-malarial spiroindolone analogue compound with promising and potent activity in clinical studies [1–3]. Spiroindolones disrupt sodium and osmotic homeostasis in *Plasmodium* species by inhibiting PfATP4, a plasma membrane Na+-ATPase [4]. Cipargamin has potent activity against all intra-erythrocytic stages of *Plasmodium falciparum* and gametocytocidal activity [4, 5].

Eight clinical trials with oral cipargamin have been conducted. A total of 152 healthy volunteers received single doses between 1 and 300 mg or 10–150 mg daily doses for 3 days, and 57 patients received single doses between 10 and 75 mg or 30 mg daily for 3 days. In addition, 8 healthy volunteers were treated in a human challenge study (induced blood stage malaria (IBSM)) at 10 mg single dose.

Cipargamin was well tolerated in healthy volunteers in Phase I trials. In Phase II trials, cipargamin showed rapid parasite clearance and fever resolution in both *P. falciparum* and *Plasmodium vivax* infections, and was well tolerated in malaria patients [3, 6, 7]. For falciparum malaria, median parasite clearance time was 12 h and parasite clearance half-life was 0.95 h following a first dose of 30 mg cipargamin [6].

In two of these trials, participants treated with cipargamin experienced transient abnormalities of liver function tests (LFTs). In a trial evaluating patients in Asia infected with *P. falciparum,* transient Grade 2–3 LFT elevations were observed in 4/11 subjects treated with a 75 mg single dose. These were as follows: one case of alanine aminotransferase (ALT) elevation up to > 5 × upper limit of normal (ULN) (Grade 3) with elevated baseline alkaline phosphatase (ALP), aspartate transaminase (AST) and ALT values; one case of ALT elevation > 5 × ULN (Grade 3) combined with Grade 2 AST elevations, with normal AST/ALT baseline levels; and, two cases of Grade 2 (> 3 × ULN) AST/ALT elevations in patients with normal baseline values. All elevations were transient and asymptomatic.

In a human challenge trial, with healthy volunteers in Australia, transient Grade 3–4 LFT elevations were observed in 3/8 subjects following a single oral dose of 10 mg cipargamin [8]. None of the cases was considered a Hy’s law case due to the predominance of unconjugated bilirubin. The sponsor terminated these trials to initiate further investigations. As both trials were uncontrolled, a possible role of cipargamin remained difficult to assess. Liver function abnormalities are common in malaria patients [9–11], and have been consistently demonstrated to occur in volunteers treated with other anti-malarials in experimental infections [12]. The sponsor conducted a detailed evaluation of the LFT elevations in the two trials, a review of hepatic safety across all trials with cipargamin, and in vitro assessments of the potential to cause hepatotoxicity [3]. This assessment concluded that the elevated LFTs in patients were most likely due to malaria. However, an effect of cipargamin could not be excluded, and therefore an additional safety dose escalation trial was to be performed prior to further clinical development.

A Phase II trial was designed to systematically address the potential hepatotoxicity of cipargamin in an actively controlled setting by comparison to the current standard of care, i.e., artemether–lumefantrine (Coartem^®^/Riamet^®^).

## Methods

### Trial design

The trial CKAE609A2202 was conducted in Mali, Gabon, Ghana, Uganda, and Rwanda. The primary objective of this multicentre, randomized, open-label, dose escalation Phase II trial was to characterize hepatic safety aspects of single and multiple ascending doses of cipargamin in adult malaria patients. Secondary objectives included overall safety and tolerability, pharmacokinetic parameters and efficacy. Here, hepatic and general safety assessments are described, while efficacy and pharmacokinetics are reported elsewhere [1].

### Patients

Eligible patients were men and women (≥ 18 years old and ≥ 45 kg) with microscopic confirmation of acute uncomplicated falciparum malaria (parasitaemia of 500 to 50,000/μL and axillary temperature ≥ 37.5 °C or oral/tympanic/rectal temperature ≥ 38.0 °C or history of fever during the previous 24 h). Exclusion criteria included mixed *Plasmodium* infections, severe malaria according to World Health Organization (WHO) criteria [13], active tuberculosis, alcohol misuse, known liver abnormalities, liver cirrhosis, known viral hepatitis B or C (patients were not screened prior to study entry), gallbladder or bile duct disease, acute or chronic pancreatitis, and abnormal LFTs (AST/ALT) > 1.5 × ULN, or AST/ALT > 1.0 and ≤ 1.5 × ULN and total bilirubin > ULN, or total bilirubin > 2 × ULN).

### Trial procedures

Open-label cipargamin treatment was administered in five sequential cohorts, using ascending single or multiple doses of cipargamin with a pause between cohorts for a review of safety data. Within each cohort, patients were randomized in parallel to treatment groups using interactive response technology (IRT). After being contacted by the investigator, the IRT assigned a randomization number (using a validated system that automated the random assignment of patient numbers to randomization numbers) that linked the patient to a treatment arm and specified a unique medication number for the study drug to be dispensed to the patient. Randomization could be suspended within a cohort if patients experienced safety events. The study or a specific treatment arm could be stopped in the event of serious safety observations especially related to hepatic safety.

**Fig. 1:**
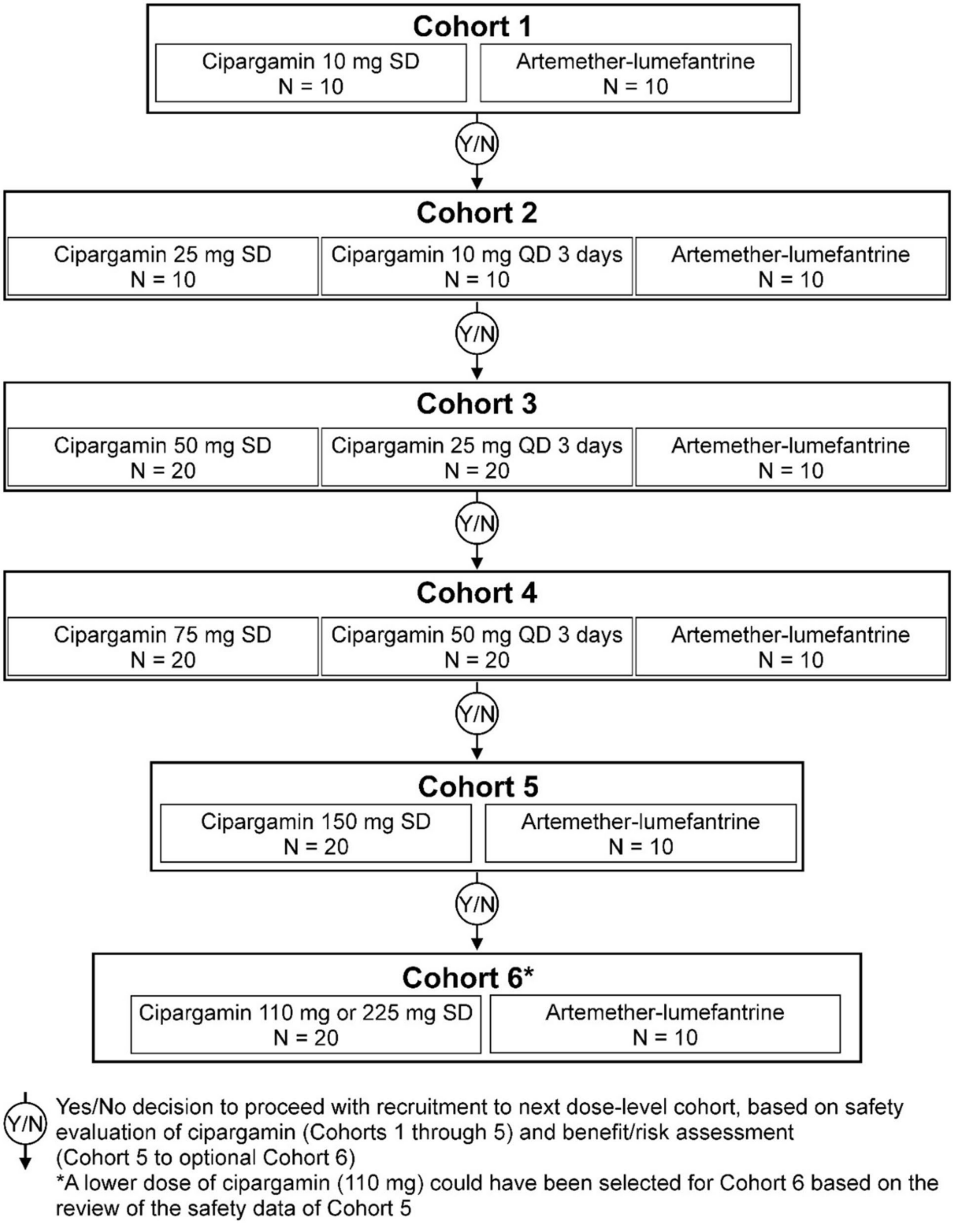
Trial design

A cipargamin starting dose of 10 mg (cohort 1) was chosen, as this was the minimum dose at which LFT elevations were observed in the IBSM trial. Following acceptable safety results in cohort 1, the dose was increased stepwise in subsequent cohorts up to a single dose of 150 mg and multiple doses of 50 mg (once daily (QD) for 3 days) according to the dosing schedule (Fig. 1), given that LFT elevations were observed at 75 mg in 4/11 patients in the previously terminated patient trial.

Originally, four cohorts were planned, with maximum cipargamin doses of 75 mg single dose and 50 mg once daily for 3 days. A protocol amendment, made when the trial was ongoing, added cohort 5 (150 mg single cipargamin dose) and an optional sixth cohort in which patients would receive 110 mg or 225 mg single doses, depending on hepatic safety in cohort 5. If, based on risk–benefit assessment, there was no added benefit expected to patients from using the 225 mg dose, the trial could be stopped after cohort 5. After completion of cohort 5, the safety review committee approved further dose escalation. However, the optional sixth cohort was not initiated as no added benefit was expected from dosing 225 mg due to expected overlapping exposures with 150 mg dose in previous cohort.

Decision criteria (provided in Additional file 1: Table S1 in the Additional Appendix) were applied according to post-baseline changes in AST and ALT levels to determine dose escalation of patients to the next cohort after notification to the safety review committee (SRC). The SRC included an independent hepatologist and a malaria expert. Following the criteria outlined in the SRC charter, formal SRC reviews were held after each cohort. SRC members reviewed the hepatic safety data and approved any further dose escalation and initiation to the following cohort.

Based on the safety pattern observed in previous trials with cipargamin, liver function parameters would be expected to peak within 14 days of treatment. Artemether–lumefantrine (80/480 mg, twice daily for 3 days) was used as an active comparator in each cohort. As the cipargamin dose in the first two cohorts was potentially sub-therapeutic, a minimum number (N = 10) of patients were included to detect possible changes in liver function parameters. In subsequent cohorts, higher numbers (N = 20) of patients were included. All patients were followed up for 28 days, with close monitoring in an inpatient setting for at least the first 3 days, followed by frequent outpatient monitoring. To be discharged, patients were required to yield two consecutive trial assessments with negative blood smears for *P. falciparum* parasites and clearance of fever. Artemether–lumefantrine was used as rescue medication in cipargamin patients meeting the protocol-specified treatment failure criteria, as this is a standard treatment for uncomplicated malaria in the countries in which the trial was conducted.

### Outcomes

The primary endpoint was at least two Common Terminology Criteria for Adverse Events (CTCAE) grades increase from baseline in ALT or AST during the 4-week trial period. Secondary endpoints reported here are standard safety and tolerability assessments of cipargamin (adverse event incidence and severity, vital signs, electrocardiography, laboratory abnormalities). Two categories of LFT abnormalities/adverse events were considered during the course of the trial: (1) liver laboratory triggers, which required repeated assessments of the abnormal laboratory parameter, and, (2) liver events, which were considered as medically significant events, i.e., were to be reported as serious adverse events which consisted of marked elevations of LFTs and/or pre-specified events: ALT or AST > 5 × ULN, ALP > 2 × ULN (in the absence of known bone pathology), total bilirubin (TBL) > 2 × baseline value, ALT or AST > 3 × ULN and International Normalized Ratio (blood clotting test, INR) > 1.5, potential Hy’s Law cases (defined as ALT or AST > 3 × ULN and TBL > 2 × ULN (mainly conjugated fraction) without a notable increase in ALP to > 2 × ULN), any clinical event of jaundice (or equivalent term), ALT or AST > 3 × ULN accompanied by (general) malaise, fatigue, abdominal pain, nausea, or vomiting, or rash with eosinophilia, any adverse event potentially indicative of a liver toxicity. These events required close observation and follow-up monitoring. Samples for LFT were collected at baseline and 24, 48, 72, 96, 168, 240, 336, 504, and 672 h post-first dose.

### Electrocardiogram

Triplicate electrocardiograms (ECGs) were performed at baseline, during and following treatments at multiple time points (4, 12, 24, and 672 h post-first dose for the single dose cohort and 4, 52, 60, 72, and 672 h post-first dose, respectively, for the three-dose cohort). Initial manual read-out was done locally in order to detect significant safety findings and allow for immediate response if needed. Additionally, all ECGs were assessed centrally by an independent and blinded (with age of patient identified) cardiologist.

### Statistical analysis

The primary (hepatic safety) and secondary (general safety) analyses were based on the safety analysis set (all randomized subjects who took at least one dose of trial drug during the treatment period). Data were summarized by cohort and treatment. For artemether–lumefantrine, data from all cohorts were pooled and summarized. As the significance of safety related results was determined by both the frequency and severity of events, there was no pre-defined hypothesis for the primary variable (occurrence of at least 2 CTCAE grades increase from baseline in ALT or AST during the 4-week trial period). The following analyses were performed for the primary variable: proportion of patients with occurrence of the primary variable by cohort and treatment group and 95% confidence interval based on the exact confidence interval (Pearson–Clopper method); and, a 2-sided Fisher exact test for each cipargamin treatment group compared to the pooled artemether–lumefantrine group. General safety variables (adverse events (AEs), laboratory parameters, vital signs, electrocardiography) were summarized by treatment.

For a cipargamin treatment group in each cohort, the probability of observing at least 2 safety events in 10 patients or 3 safety events in 20 patients is at least 80% if the true event rate is ≥ 30%, and less than 5% if the true event rate is ≤ 3%. Given the sample size of 10 to 20 patients per treatment group within a cohort, the power to detect treatment difference is low even with large treatment differences using a 2-sided statistical significance level of 5%. If the true rate is 50% for a cipargamin treatment group and 3% for the artemether–lumefantrine treatment group, the power to detect between treatment difference is about 80% using a 2-sided test at the 20% significance level for n = 10 per treatment group or at the 10% significance level for n = 20 for a cipargamin treatment group and 10 for the artemether–lumefantrine treatment group. With 4 cohorts for a total of 40 patients treated with artemether–lumefantrine group, the power to detect treatment difference using 2-sided test at the 5% significance level is 77% and 91% for n = 10 and 20 patients treated with cipargamin, respectively, if the true rate is 40% for cipargamin and 5% for the artemether–lumefantrine.

## Results

### Patients

The trial started on 16 November, 2017 and completed on 23 November, 2019. A total of 188 patients were randomized (Additional file 1: Fig. S1) at centres in Mali (11), Uganda (58), Ghana (29), Gabon (16), and Rwanda (74). Due to seasonal and operational aspects, not all countries contributed equally to all cohorts. Two cipargamin patients were randomized but not treated and were excluded from the analyses. One patient (cipargamin 10 mg single dose) discontinued immediately after treatment. One cipargamin and one artemether–lumefantrine patient did not complete follow-up.

Patient demographics were comparable across treatment groups and consistent with the intended target population as specified in the inclusion criteria. Patients were 18 to 61 years old; most patients (approximately 62%) were male. Cohorts were balanced in terms of baseline characteristics, except for baseline parasite counts, which tended to be higher in cohorts 4 and 5 (mean > 13,600/µL) than in cohorts 1 to 3 (means ranging from 3297 to 9884/µL; Additional file 1: Table S2).

### Hepatic safety assessments

**Fig. 2:**
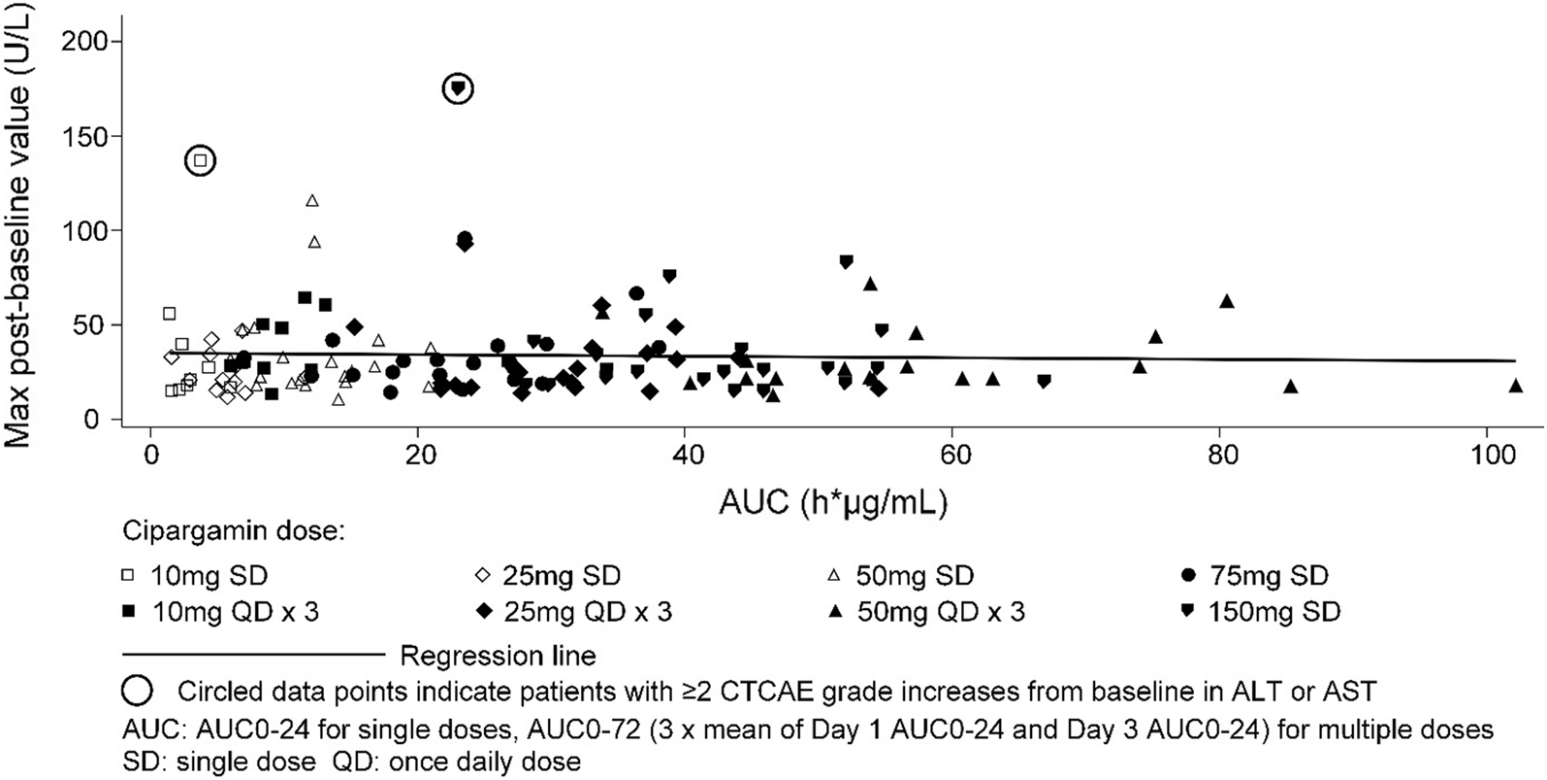
Scatter plot of max post-baseline ALT absolute value *versus* cipargamin AUC (area under the curve) by treatment group (Safety set)

Overall, 2/135 (1.48%) patients treated with cipargamin had at least two CTCAE grade increases from baseline in ALT or AST compared to 2/51 (3.92%) patients treated with artemether–lumefantrine (detailed LFT results for these 4 cases are available in supplement). There was no significant difference in the proportion of patients with two CTCAE grade increases between any cipargamin cohort and the pooled artemether–lumefantrine group (Table 1). No patient experienced a Grade 4 LFT event. There was no obvious relationship between cipargamin dose or exposure, which was approximately dose-proportional, and hepatic safety in terms of maximum ALT values post-baseline (Fig. 2).

**Table 1:** Proportion of patients with at least 2 CTCAE grades increase from baseline in ALT or AST (Safety set)

Treatment group	At least 2 CTCAE grades increase in AST or ALT			
	m	n (%)	95% Pearson–Clopper CI (%)	2-sided p-value^a^
Cipargamin 10 mg single dose (N = 10)	9	1 (11.1)	(0.3,48.2)	0.391
Cipargamin 10 mg QD 3 days (N = 10)	10	0 (0.0)	(0.0,30.8)	1
Cipargamin 25 mg single dose (N = 12)	12	0 (0.0)	(0.0,26.5)	1
Cipargamin 25 mg QD 3 days (N = 20)	20	0 (0.0)	(0.0,16.8)	1
Cipargamin 50 mg single dose (N = 21)	21	0 (0.0)	(0.0,16.1)	1
Cipargamin 50 mg QD 3 days (N = 19)	19	0 (0.0)	(0.0,17.6)	1
Cipargamin 75 mg single dose (N = 21)	21	0 (0.0)	(0.0,16.1)	1
Cipargamin 150 mg single dose (N = 22)	22	1 (4.5)	(0.1,22.8)	1
Pooled artemether–lumefantrine (N = 51)	51	2 (3.9)	(0.5,13.5)	–

*N* number of patients in the respective treatment group, *m* number of patients with baseline and at least one post-baseline assessment for either ALT or AST, *n* number of patients who meet the criterion. *%* 100*n/m, *QD* once daily

^a^2-sided p-value results from Fisher exact test for each cipargamin treatment group compared to pooled artemether–lumefantrine

One patient treated with 10 mg single-dose cipargamin had an asymptomatic increase in ALT from normal levels at baseline to Grade 2 (137 U/L, approximately 3 × ULN) by day 8, with normalization by day 29. AST and TBL remained within the normal range.

**Fig. 3:**
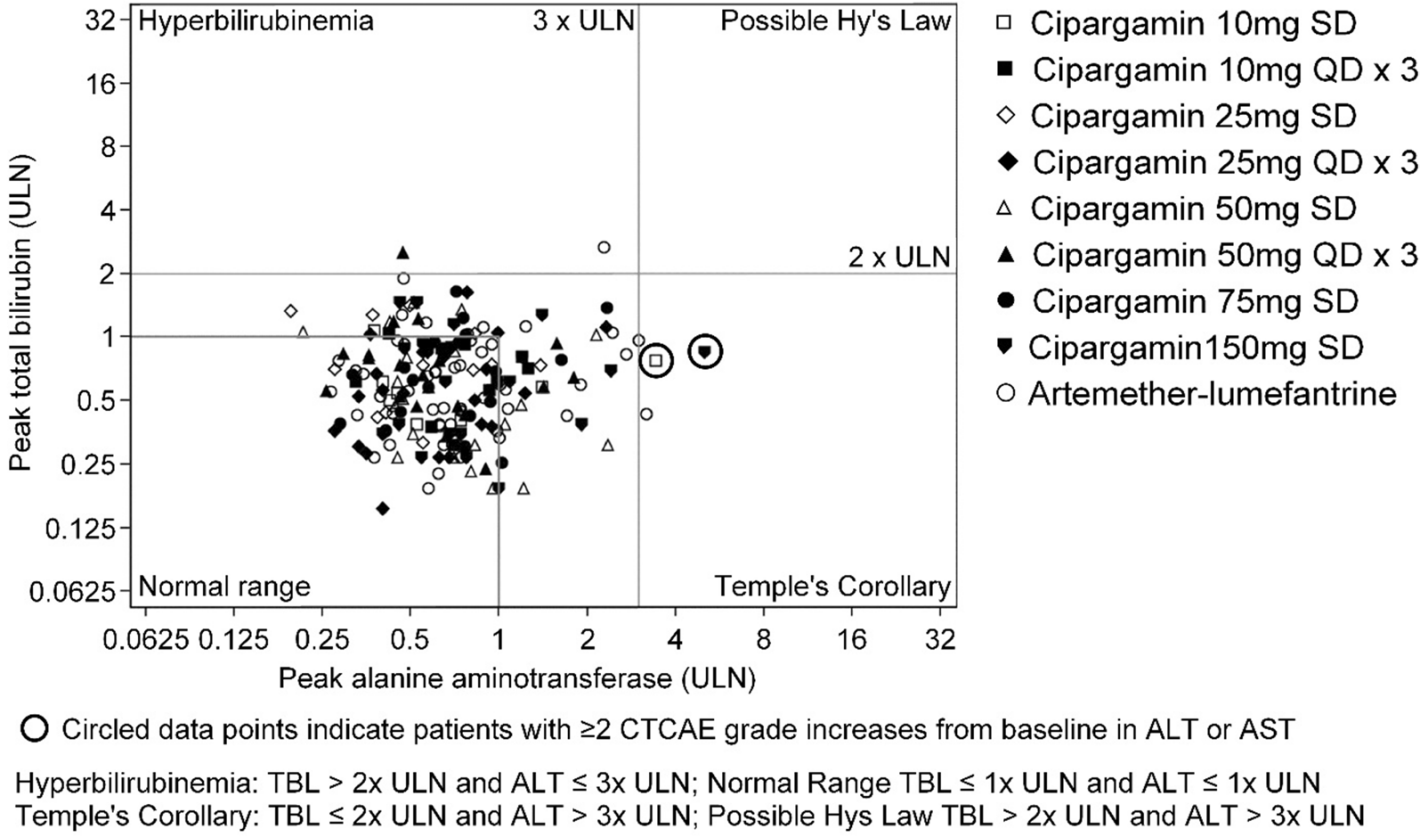
eDISH (evaluation of drug-induced serious hepatotoxicity) of ALT against bilirubin by cohort and treatment (Safety set)

One patient treated with 150 mg single-dose cipargamin had an asymptomatic increase in ALT from Grade 1 at baseline (36 U/L, normal range 7–35 U/L) to Grade 3 (176 U/L, > 5 × ULN) on day 4, returning to Grade 1 on day 11 and normalized by day 15. Per-protocol, this event was reported as a serious adverse event. Cipargamin exposure for this patient was the lowest in the cohort (Fig. 2). This patient’s AST levels were > 3 × ULN on day 2 and normalized by day 8; TBL remained within the normal range (Fig. 3).

Total bilirubin exceeded 2 × ULN in one patient treated with cipargamin and in one artemether–lumefantrine patient. TBL normalized in both patients before day 29. Hy’s law criteria were not met for any patient (Fig. 3, Additional file 1: Table S3) and there was no event of jaundice. ALP exceeded 2 × ULN in an 18-year-old male patient treated with 75 mg single-dose cipargamin. ALP was high at baseline, worsened to > 2 × ULN at day 2 and was still elevated at day 29.

To evaluate potential effects of anti-malarial drug combinations on hepatic safety, a sub-group of patients receiving cipargamin for the initial malaria infection and artemether–lumefantrine as rescue medication for treatment of a recurrent malaria infection was defined. This sub-group included only patients for whom at least one follow-up visit was conducted a week after initiation of a full course of artemether–lumefantrine. All patients receiving cipargamin on day 1 and artemether–lumefantrine before or at visit day 22 qualified for this analysis. In this small sub-group (N = 23), 82.6% (19/23) of patients experienced no AST or ALT elevation, while 17.4% (4/23) had a Grade 1 ALT or AST increase. There were no Grade 2 or 3 events. In comparison, 74.1 (100/135) and 64.7% (33/51) of patients treated with cipargamin or artemether–lumefantrine alone had no ALT or AST grade elevation.

### General safety and tolerability

All cipargamin doses were tolerated well. There were no discontinuations due to AEss. AE rates were similar across treatment groups, with no obvious relationship to cipargamin dose. Gender did not appear to impact the incidence of AEs. Most AEs were considered not related to the study drug. Nineteen out of 135 (14.1%) patients treated with cipargamin and 5/51 (9.8%) patients treated with artemether–lumefantrine had at least one AE suspected to be related to the study drug. None of these events was considered serious except for one event of increased ALT, as described above.

The most common AEs were disease-related signs and symptoms, including headache, malaria and treatment failure. Malaria reported as an AE was defined as worsening malaria symptoms or recrudescence/re-infection. AEs of Grade 3–4 severity were reported for four cipargamin patients (Grade 3 leukopaenia, Grade 4 thrombocytopaenia, Grade 3 ALT increase, Grade 3 hypomagnesemia) and two artemether–lumefantrine patients (Grade 3 hyperbilirubinaemia and Grade 3 gamma-glutamyl transpeptidase; Grade 3 thrombocytopaenia). These events were transient, not dose-related and resolved without treatment. Haematological abnormalities, including anaemia and thrombocytopaenia, of varying severity, are common in malaria patients [14].

Hepatic AEs were reported in 10/135 (7.4%) patients in cipargamin groups as compared to 6/51 (11.8%) in the pooled artemether–lumefantrine group.

Five non-fatal serious AEs were reported; four in cipargamin patients (Grade 2 bilirubin increase from day 15 to day 21 (50 mg QD for 3 days), Grade 2 ALP increase from day 2 (75 mg single dose), Grade 3 ALT increase from day 4 to day 11 (150 mg single dose), Grade 4 thrombocytopaenia from day 9 to day 35 (75 mg single dose) and one in an artemether–lumefantrine patient (Grade 2 bilirubin increase from day 2 to day 8). Four of these events met protocol-defined serious AE criteria for elevations of LFTs. All were asymptomatic, transient and resolved within a few days without treatment. None was suspected to be related to the trial drug except for the Grade 3 ALT elevation (Table 2).

**Table 2:** Summary of most common adverse events (≥ 20% of patients in any group) and serious adverse events, regardless of trial treatment relationship, by preferred term and treatment (Safety set)

	Cipargamin 10 mg single dose N = 10 n (%)	Cipargamin 10 mg QD/3 days N = 10 n (%)	Cipargamin 25 mg single dose N = 12 n (%)	Cipargamin 25 mg QD/3 days N = 20 n (%)	Cipargamin 50 mg single dose N = 21 n (%)	Cipargamin 50 mg QD/3 days N = 19 n (%)	Cipargamin 75 mg single dose N = 21 n (%)	Cipargamin 150 mg single dose N = 22 n (%)	Pooled artemether–lumefantrine N = 51 n (%)
Patients with adverse event(s)	9 (90.0)	8 (80.0)	10 (83.3)	14 (70.0)	14 (66.7)	16 (84.2)	19 (90.5)	13 (59.1)	33 (64.7)
Most common adverse events									
Malaria	1 (10.0)	0	2 (16.7)	4 (20.0)	4 (19.0)	5 (26.3)	5 (23.8)	9 (40.9)	1 (2.0)
Headache	3 (30.0)	5 (50.0)	1 (8.3)	1 (5.0)	2 (9.5)	2 (10.5)	5 (23.8)	0	9 (17.6)
Treatment failure	0	1 (10.0)	3 (25.0)	1 (5.0)	0	1 (5.3)	0	0	2 (3.9)
Patients with serious adverse events	0	0	0	0	0	1 (5.3)	2 (9.5)	1 (4.5)	1 (2.0)
Thrombocytopenia	0	0	0	0	0	0	1 (4.8)	0	0
ALT increased	0	0	0	0	0	0	0	1 (4.5)	0
Blood ALP increased	0	0	0	0	0	0	1 (4.8)	0	0
Blood bilirubin increased	0	0	0	0	0	1 (5.3)			1 (2.0)

*QD* once daily, *ALT* alanine aminotransferase, *ALP* alkaline phosphatase

### Electrocardiography

Minor elevations (> 30 to ≤ 60 ms) in QT interval corrected by Fridericia’s formula (QTcF) occurred in all treatment groups, with frequencies from 9.1 to 36.8% in the cipargamin treatment groups and 27.5% in the pooled Coartem controls. Asymptomatic increases of > 60 ms were noted on day 29 in two patients treated with cipargamin 25 mg QD for 3 days, and on day 2 in one patient treated with 75 mg single-dose cipargamin. These did not appear to be related to cipargamin exposure. No patient had QTcF > 500 ms. An increase of > 25% in heart rate (> 100 bpm) was reported for one patient in the cipargamin 150 mg single dose group and in one artemether–lumefantrine patient; while a heart rate decrease of > 25% (< 50 bpm) was reported for one patient each in the cipargamin 50 mg single-dose, 75 mg single-dose, and 25 mg multiple-dose groups. None of the abnormalities was considered clinically significant by the investigators, and hence not reported as AEs (Additional file 1: Table S4).

### Vital signs

No meaningful change from baseline was observed in any of the vital signs across cohorts or treatment groups. No patients experienced a clinically notably high systolic blood pressure (SBP) (≥ 180 mmHg or increase from baseline by ≥ 20 mmHg) or diastolic BP (≥ 105 mmHg or increase from baseline by ≥ 15 mmHg). Clinically notably low SBP (≤ 90 mmHg or decrease from baseline by ≥ 20 mmHg) or DBP (≤ 50 mmHg or decrease from baseline by ≥ 15 mmHg) were observed in two patients treated with a single 75 mg dose of cipargamin compared to four patients treated with artemether–lumefantrine.

## Discussion

There is an urgent need for the development of new non-artemisinin-based anti-malarial drugs. Cipargamin is a promising new anti-malarial that showed rapid parasite clearance in Phase II trials. Some transient, mainly asymptomatic, LFT elevations occurred in two previous trials with cipargamin; one in patients with *P. falciparum* and one IBSM trial. No significant LFT elevations were identified in healthy volunteers given a single dose of up to 300 mg [7].

The trial reported here evaluated the hepatic safety of cipargamin in adult patients with uncomplicated falciparum malaria, applying a dose escalation design, using 10 mg to 150 mg single doses and 10 mg to 50 mg QD for three days, and a control arm. The low rate of LFT abnormalities in patients treated with cipargamin was comparable to that in artemether–lumefantrine patients. There was no obvious relationship between LFT abnormalities and cipargamin dose or exposure. Exposure to cipargamin in this trial was higher or comparable to previous trials [1], with no evidence of an increase in hepatic events. In addition, patients receiving both cipargamin and artemether–lumefantrine during the study did not show an increased rate of LFT abnormalities.

The SRC did not observe any serious hepatic safety concerns after cohorts 1, 3, 4, and 5, and approved further dose escalation after each review. No further escalation was made as no increased benefit was expected for the patients, so the trial was considered complete after completion of cohort 5.

As cipargamin will be further developed as a combination treatment for uncomplicated malaria, good tolerability, including liver safety, when administered with other anti-malarials is important. The current trial included only adults from sub-Saharan African countries with falciparum malaria. Comparison with the two previous cipargamin trials (in Asia and Australia) therefore requires caution as no clear conclusion can be drawn as to whether hepatic safety and potential immunity to malaria would differ with ethnicity, patient age and or geography. The differences in baseline parasite counts in the current trial may complicate comparisons between cipargamin doses and between cipargamin and artemether–lumefantrine in terms of safety. In addition, patients with known liver abnormalities or disease were excluded from the current trial, thereby the effects of cipargamin on those with pre-existing liver abnormalities cannot be determined from this trial.

Abnormalities in liver function parameters are common in malaria patients and have also been observed in volunteers with experimental infection. In IBSM studies, LFT elevations appear to be specific to the experimental model as similar results were seen with multiple tested compounds of different chemical classes [9, 12, 15]. Liver enzyme elevations in IBSM studies are expected to be due to numerous factors resulting from malaria infection in healthy subjects such as parasite density, acute inflammation, oxidative stress, effective parasite clearance, or by participant-specific risk factors, acetaminophen administration, increasing susceptibility to effects on the liver from acetaminophen or study drugs [12]. Based on the observations, changes to the design of IBSM studies with new chemical entities are proposed to minimize hepatotoxicity risk in study participants.

In malaria patients, changes in LFT results are likely to be an inherent, although variable, aspect of the disease; individual-specific factors may confer particular susceptibility to hepatocyte injury. These abnormalities are transient and may vary widely in severity (up to > 25 ULN) [15]. Elevated serum liver enzyme levels in acute malaria are associated with changes in hepatic function resulting from histopathological changes such as hyperplastic Kupffer cells, fatty change, portal tract inflammation, cholestasis, liver cell necrosis, sequestration of erythrocytes, and deposition of haemozoin pigment [10, 11]. In a recent observational study investigating LFT abnormalities in patients with imported uncomplicated malaria, reversible liver injury, predominantly ALT and AST elevations, was found to be a common feature, occurring regardless of the drug regimen. There were significant associations between LFT elevations and parasite load, inflammatory markers and a reduced expression of oxidative stress markers [9].

## Conclusions

Cipargamin was well tolerated in this study without any hepatic safety concerns at a wider dose range and higher exposures than previous studies. This trial, the first conducted in Africa and the largest carried out to date with cipargamin, showed the hepatic safety of cipargamin was comparable to that of artemether–lumefantrine in adult patients with uncomplicated falciparum malaria. This indicates that elevations in liver function tests observed in previous trials with cipargamin are likely to be malaria-related in patients and confounded by the experimental model in the human challenge trial and lack of an active control. This study paves the way for the further development of this novel anti-malarial. In addition to the good tolerability of cipargamin, this study has confirmed its potency and rapid onset of parasiticidal activity with parasite clearance times of around 8 h for doses of 50 mg or higher [1]. Furthermore, cipargamin is potent against artemisinin-resistant parasites including the R561H mutant, which is spreading in Rwanda [2]. However, when used as monotherapy, treatment-emerging mutations were detected in patients experiencing recrudescences [1]. Cipargamin will be developed in fixed-dose combination with a partner drug and liver safety aspects remain to be considered for selection of partner compounds and monitored in future clinical trials. A cipargamin-based combination with a suitable partner drug with a high barrier to resistance will address the urgent medical need for the development of new non-artemisinin-based anti-malarial drugs.

This example of monitoring liver safety of clinical candidates in a disease that itself is causing increases in liver function tests and the challenge of dissecting the potential drug related causality from disease background should inform future development programmes in related areas.

## Supplementary Information

**Additional file 1.** Supplement Hepatic safety and tolerability of cipargamin (KAE609).

## Data Availability

ClinicalTrials.gov number: NCT03334747.

## Abbreviations

AE: Adverse events
ALP: Alkaline phosphatase
ALT: Alanine aminotransferase
AST: Aspartate aminotransferase
AUC: Area under the curve
CTCAE: Common Terminology Criteria for Adverse Events
DBP: Diastolic blood pressure
Hg: Mercury
IBSM: Induced blood stage malaria
INR: International normalized ratio (blood clotting test)
IRT: Interactive response technology
QD: Once daily
LFT: Liver function test
SBP: Systolic blood pressure
SD: single dose
SRC: Safety Review Committee
TBL: Total bilirubin
ULN: Upper limit of normal
