# Supplementary material for: Hepatic safety and tolerability of cipargamin (KAE609), in adult patients with Plasmodium falciparum malaria: a randomized, phase II, controlled, dose-escalation trial in sub-Saharan Africa
Source: Malar J. 2021 Dec 20;20:478. doi: 10.1186/s12936-021-04009-1 (PMC8686384; doi:10.1186/s12936-021-04009-1)

**Supplementary Material to**: **Hepatic safety and tolerability of cipargamin (KAE609), in adult patients with *Plasmodium falciparum* malaria: a randomized, phase II, controlled, dose-escalation trial in sub-Saharan Africa**

**Contents**

The CKAE609A2202 study group 2

Figure S1. CONSORT patient flow 4

Table S1. Decision criteria for dose escalation based on LFT results with cipargamin 5

Table S2. Summary of baseline demographics and disease characteristics (Randomized set) 6

Table S3. Newly occurring liver enzyme abnormalities any time post-baseline (Safety set) 8

Table S4. Summary of patients with designated changes in QTcF and HR intervals (Safety set) 9

LFT changes for 4 cases with 2 CTCAE grade increase in AST/ALT 10

**The CKAE609A2202 study group**

In addition to the authors, the following study group members were all closely involved with the implementation, conduct and oversight of the clinical trial. Study centers are listed in alphabetical order.

**Center for Family Health Research, Kigali, Rwanda (author: Etienne Karita)**

Julien Nyombayire

Rosine Ingabire

Jean Bizimana

**Centre de Recherches Médicales en Lambaréné,, Lambaréné, Gabon (author: Martin P. Grobusch)**

Ghyslain Mombo-Ngoma

Rella Zoleko Manego

Peter Kremsner

**Infectious Diseases Research Collaboration, Busia, Uganda (author: Adoke Yeka)**

Afizi Kibuuka

Kenneth Kalamba

Juliet Imokol

Annet Nalwoga

**Joint Clinical Research Centre, Kampala, Uganda (author: Henry Mugerwa)**

Esther Kabaswaga

Margret Ndagire

Peter Erimu

Dridah Nakiboneka

**Kabwohe Clinical Research Center, Sheema, Uganda (author: Stephen Asiimwe)**

Anacret Byamukama

Edna Brenda Tindimwebwa

Edgar Nduho Ngarame

John Paul Ninkusiima

**Kintampo Health Research Centre, Kintampo North Municipality, Ghana (author: Kwaku Poku Asante)**

Seyram Kaali

Prince Agyapong Darko

Samuel Harrison

Elvis Wilson

**Malaria Research and Training Center, Faculty of Pharmacy, University of Science, Techniques and Technologies of Bamako, Bamako, Mali (author: Bakary Fofana)**

Mohamed Lamine Alhousseini

Amadou Bamadio

Souleymane Dama

**Navrongo Health Research Centre, Ghana Health Service, Navrongo, Ghana (author: Abraham Oduro)**

Thomas Anyorigiya

Michael Kaburise

Jonas Kulariba

Mathilda Tsifodze

**Rinda Ubuzima, Kigali, Rwanda (author: Gilles Ndayisaba)**

Jean Claude Ndagijimana

Marie Michelle Umulisa

Rosette Busasa

Lambert Mwambarangwe

**University Clinical Research Center, University of Sciences, Techniques and Technology of Bamako*,* Bamako, Mali (author: Seydou Doumbia)**

Mahamadou Diakite

Sory Ibrahima Diawara

Drissa Konate

Mariam Coulibaly

**Figure S1. CONSORT patient flow**

Randomized
N = 188

Cipargamin
50 mg SD

N = 22

Treated

N = 21

Not treated
Technical problems

N = 1

Completed treatment period

N = 21

Completed follow-up
period

N = 21

*Pooled artemether-lumefantrine groups from all cohorts

Completed follow-up
period

N = 10

Completed follow-up period

N = 9

Discontinued

N = 1
Withdrew
consent

Completed treatment period

N = 9

Completed treatment
period

N = 10

Treated

N = 10

Treated

N = 10

Cipargamin
10 mg QD 3 days

N = 10

Cipargamin
10 mg SD

N = 10

Artemether-lumefantrine

N = 51*

Treated

N = 51

Completed treatment period

N = 51

Completed follow-up
period

N = 50

Discontinued

N = 1
Patient/
guardian decision

Cipargamin
150 mg SD

N = 22

Treated

N = 22

Completed treatment period

N = 22

Completed follow-up
period

N = 22

Cipargamin
75 mg SD

N = 21

Treated

N = 21

Completed treatment period

N = 21

Completed follow-up
period

N = 20

Discontinued

N = 1
Patient/
guardian decision

Cipargamin
50 mg QD 3 days

N = 20

Treated

N = 19

Not treated
Withdrew consent

N = 1

Completed treatment
period

N = 19

Completed follow-up
period

N = 19

Cipargamin
25 mg QD 3 days

N = 20

Treated

N = 20

Completed treatment
period

N = 20

Completed
follow-up
period

N = 20

Cipargamin
25 mg SD

N = 12

Treated

N = 12

Completed treatment period

N = 12

Completed follow-up
period

N = 12

**Table S1. Decision criteria for dose escalation based on LFT results with cipargamin**

| **LFT parameter** | **Baseline** | **Maximum post baseline value** | **Decision to escalate to next cohort** |
| --- | --- | --- | --- |
| ALT/AST | Within ULN | < 2 times ULN (from **Day 1 to Day 15**) | Escalate to next cohort after notification to safety review committee, after all the patients in the cohort have been followed for at least 14 days post treatment (Study Day 15). |
|  | > 1 to ≤ 1.5 ULN | < 2 times baseline (from **Day 1 to Day 15**) |  |
| ALT/AST | Within ULN | ≥ 2 to < 3 times ULN (from **Day 1 to Day 15**) | Escalate to next cohort based on review of liver safety (and any other relevant data) by safety review committee, after all the patients in the cohort have been followed for at least 28 days post treatment (**Study Day 29**) |
|  | > 1 to ≤ 1.5 ULN | ≥ 2 to < 3 times baseline (from **Day 1 to Day 15**) |  |
| ALT/AST | Any baseline value | ≥ 2 Grade (CTCAE grades) increase from baseline (**at any time point during the study**) in: 2 patients in a 10-patient cohort (Cohorts 1 and 2)  or  3 patients in a 20-patient cohort (Cohorts 3, 4, 5, and 6) | Suspend recruitment and initiate review of liver safety (and any other relevant data) by safety review committee.  Any further progression of the study is based on the decision by the safety review committee |
| ALT: alanine aminotransferase, AST: aspartate aminotransferase, ULN: upper limit of the normal range | | | |

**Table S2. Summary of baseline demographics and disease characteristics (Randomized set)**

| **Characteristic** | **Cipargamin 10 mg single dose N=10** | **Cipargamin 10 mg QD/3 days N=10** | **Cipargamin 25 mg single dose N=12** | **Cipargamin 25 mg QD/3 days N=20** | **Cipargamin 50 mg single dose N=22** | **Cipargamin 50 mg  QD/3 days N=20** | **Cipargamin 75 mg single dose N=21** | **Cipargamin 150 mg single dose N=22** | **Pooled Artemether-lumefantrine N=51** |
| --- | --- | --- | --- | --- | --- | --- | --- | --- | --- |
| **Age (years)** |  |  |  |  |  |  |  |  |  |
| Mean (SD) | 31.5 (10.48) | 35.4 (13.25) | 33.0 (14.96) | 31.9 (10.67) | 33.9 (12.56) | 26.4 (7.18) | 28.2 (10.25) | 30.6 (12.57) | 26.2 (9.07) |
| Median | 33.5 | 35.5 | 33.5 | 30.5 | 28.0 | 25.0 | 26.0 | 24.0 | 23.0 |
| Min-Max | 18-48 | 18-55 | 18-60 | 18-50 | 18-58 | 18-46 | 18-61 | 18-58 | 18-53 |
| **Age group - n (%)** |  |  |  |  |  |  |  |  |  |
| < 65 years | 10 (100) | 10 (100) | 12 (100) | 20 (100) | 22 (100) | 20 (100) | 21 (100) | 22 (100) | 51 (100) |
| **Sex - n (%)** |  |  |  |  |  |  |  |  |  |
| Male | 8 (80.0) | 4 (40.0) | 4 (33.3) | 9 (45.0) | 13 (59.1) | 12 (60.0) | 16 (76.2) | 16 (72.7) | 33 (64.7) |
| Female | 2 (20.0) | 6 (60.0) | 8 (66.7) | 11 (55.0) | 9 (40.9) | 8 (40.0) | 5 (23.8) | 6 (27.3) | 18 (35.3) |
| **BMI (kg/m2)** |  |  |  |  |  |  |  |  |  |
| Mean (SD) | 20.22 (2.815) | 23.55 (4.263) | 23.86 (4.436) | 22.57 (3.743) | 23.71 (4.323) | 21.81 (2.718) | 21.21 (2.133) | 22.35 (2.984) | 22.30 (3.503) |
| Median | 19.64 | 23.14 | 22.98 | 21.34 | 22.15 | 21.32 | 21.14 | 22.01 | 21.96 |
| Min-Max | 17.1-25.5 | 18.3-30.8 | 17.8-32.1 | 17.4-32.5 | 17.8-35.8 | 18.3-29.4 | 17.8-26.1 | 17.2-30.9 | 17.4-36.8 |
| **BMI - n (%)** |  |  |  |  |  |  |  |  |  |
| < 16 kg/m2 | 0 | 0 | 0 | 0 | 0 | 0 | 0 | 0 | 0 |
| 16 - 25 kg/m2 | 9 (90.0) | 7 (70.0) | 8 (66.7) | 16 (80.0) | 15 (68.2) | 18 (90.0) | 19 (90.5) | 19 (86.4) | 45 (88.2) |
| > 25 kg/m2 | 1 (10.0) | 3 (30.0) | 4 (33.3) | 4 (20.0) | 7 (31.8) | 2 (10.0) | 2 (9.5) | 3 (13.6) | 6 (11.8) |
| **Body temperature (°C)** |  |  |  |  |  |  |  |  |  |
| n | 10 | 10 | 12 | 20 | 22 | 20 | 21 | 22 | 51 |
| Mean (SD) | 37.30 (0.872) | 36.98 (0.973) | 36.41 (0.571) | 37.03 (0.585) | 36.88 (0.874) | 36.76 (1.019) | 37.00 (1.133) | 36.80 (1.039) | 37.18 (1.045) |
| Median | 37.35 | 36.60 | 36.25 | 37.00 | 36.60 | 36.60 | 36.80 | 36.45 | 37.00 |
| Min-Max | 36.2-39.3 | 36.1-39.0 | 35.6-37.4 | 36.3-38.6 | 35.9-39.3 | 35.2-38.6 | 35.2-39.9 | 35.0-39.2 | 35.6-39.7 |

| **Characteristic** | **Cipargamin 10 mg single dose N=10** | **Cipargamin 10 mg QD/3 days N=10** | **Cipargamin 25 mg single dose N=12** | **Cipargamin 25 mg QD/3 days N=20** | **Cipargamin 50 mg single dose N=21** | **Cipargamin 50 mg  QD/3 days N=19** | **Cipargamin 75 mg single dose N=21** | **Cipargamin 150 mg single dose N=22** | **Pooled Artemether-lumefantrine N=51** |
| --- | --- | --- | --- | --- | --- | --- | --- | --- | --- |
| **Body temperature (axillary) category (°C)**  **- n (%)** |  |  |  |  |  |  |  |  |  |
| < 37.5 | 1 (10.0) | 7 (70.0) | 11 (91.7) | 11 (55.0) | 11 (50.0) | 15 (75.0) | 15 (71.4) | 17 (77.3) | 27 (52.9) |
| 37.5 - < 39 | 0 | 1 (10.0) | 0 | 1 (5.0) | 2 (9.1) | 5 (25.0) | 4 (19.0) | 4 (18.2) | 9 (17.6) |
| ≥ 39 | 0 | 1 (10.0) | 0 | 0 | 1 (4.5) | 0 | 2 (9.5) | 1 (4.5) | 5 (9.8) |
| ***P. falciparum* density (/**µ**L)** |  |  |  |  |  |  |  |  |  |
| Mean | 3297.4 | 9883.5 | 6905.8 | 8736.6 | 8399.7 | 16992.8 | 13634.3 | 14820.8 | 14753.9 |
| (SD) | (4263.87) | (14537.19) | (10520.48) | (8817.24) | (7247.61) | (9758.97) | (12698.30) | (12633.46) | (12129.56) |
| Median | 850.0 | 1736.0 | 2406.0 | 6430.0 | 5641.5 | 15696.5 | 8190.0 | 9455.5 | 12800.0 |
| Min-Max | 502-11105 | 617-46600 | 673-37681 | 725-34580 | 639-29251 | 1654-32471 | 568-39663 | 1163-42955 | 608-47363 |
| ***P. falciparum* density (/**µ**L) categories - n (%)** |  |  |  |  |  |  |  |  |  |
| < 500 | 0 | 0 | 0 | 0 | 0 | 0 | 0 | 0 | 0 |
| 500 - < 2,000 | 7 (70.0) | 6 (60.0) | 6 (50.0) | 4 (20.0) | 5 (22.7) | 1 (5.0) | 1 (4.8) | 1 (4.5) | 13 (25.5) |
| 2,000 - < 5,000 | 0 | 0 | 1 (8.3) | 5 (25.0) | 5 (22.7) | 2 (10.0) | 3 (14.3) | 5 (22.7) | 3 (5.9) |
| 5,000 - < 15,000 | 3 (30.0) | 2 (20.0) | 4 (33.3) | 7 (35.0) | 8 (36.4) | 5 (25.0) | 10 (47.6) | 8 (36.4) | 12 (23.5) |
| 15,000 - < 50,000 | 0 | 2 (20.0) | 1 (8.3) | 4 (20.0) | 4 (18.2) | 12 (60.0) | 7 (33.3) | 8 (36.4) | 23 (45.1) |
| ≥ 50,000 | 0 | 0 | 0 | 0 | 0 | 0 | 0 | 0 | 0 |
| QD: once daily, SD: standard deviation | | | | | | | | | |

**Table S3. Newly occurring liver enzyme abnormalities any time post-baseline (Safety set)**

|  | **Cipargamin 10 mg single dose**  **N=10 n/m (%)** | **Cipargamin 10 mg**  **QD/3 days**  **N=10 n/m (%)** | **Cipargamin 25 mg single dose**  **N=12 n/m (%)** | **Cipargamin 25 mg**  **QD/3 days**  **N=20 n/m (%)** | **Cipargamin 50 mg single dose**  **N=21 n/m (%)** | **Cipargamin 50 mg  QD/3 days**  **N=19 n/m (%)** | **Cipargamin 75 mg single dose**  **N=21 n/m (%)** | **Cipargamin 150 mg single dose**  **N=22 n/m (%)** | **Pooled Artemether-lumefantrine**  **N=51 n/m (%)** |
| --- | --- | --- | --- | --- | --- | --- | --- | --- | --- |
| ALT > 3x ULN | 1/9 (11.1) | 0/10 | 0/12 | 0/20 | 0/21 | 0/19 | 0/21 | 1/22 (4.5) | 1/51 (2.0) |
| ALT > 5x ULN | 0/9 | 0/10 | 0/12 | 0/20 | 0/21 | 0/19 | 0/21 | 1/22 (4.5) | 0/51 |
| ALT > 8x ULN | 0/9 | 0/10 | 0/12 | 0/20 | 0/21 | 0/19 | 0/21 | 0/22 | 0/51 |
| AST > 3x ULN | 0/9 | 0/10 | 0/12 | 0/20 | 0/21 | 0/19 | 0/21 | 1/22 (4.5) | 1/51 (2.0) |
| AST > 5x ULN | 0/9 | 0/10 | 0/12 | 0/20 | 0/21 | 0/19 | 0/21 | 0/22 | 0/51 |
| ALT or AST > 3x ULN | 1/9 (11.1) | 0/10 | 0/12 | 0/20 | 0/21 | 0/19 | 0/21 | 1/22 (4.5) | 2/51 (3.9) |
| ALT or AST > 5x ULN | 0/9 | 0/10 | 0/12 | 0/20 | 0/21 | 0/19 | 0/21 | 1/22 (4.5) | 0/51 |
| ALT or AST > 3x ULN & TBL > 2x ULN | 0/9 | 0/10 | 0/12 | 0/20 | 0/21 | 0/19 | 0/21 | 0/22 | 0/51 |
| ALP > 2x ULN | 0/8 | 0/10 | 0/11 | 0/20 | 0/21 | 0/19 | 1/21 (4.8) | 0/22 | 0/51 |
| ALP > 3x ULN | 0/8 | 0/10 | 0/11 | 0/20 | 0/21 | 0/19 | 0/21 | 0/22 | 0/51 |
| TBL > 1.5x ULN | 0/9 | 0/10 | 0/11 | 1/20 (5.0) | 0/21 | 0/17 | 1/17 (5.9) | 0/20 | 1/44 (2.3) |
| TBL > 2x ULN | 0/9 | 0/10 | 0/12 | 0/20 | 0/21 | 1/19 (5.3) | 0/21 | 0/22 | 1/50 (2.0) |
| ALP > 3x ULN & TBL > 2x ULN | 0/9 | 0/10 | 0/12 | 0/20 | 0/21 | 0/19 | 0/21 | 0/22 | 0/51 |
| ALT or AST > 3x ULN & TBL > 2x ULN & ALP < 2x ULN (Hy's Law) | 0/9 | 0/10 | 0/12 | 0/20 | 0/21 | 0/19 | 0/21 | 0/22 | 0/51 |

Newly occurring: Patients not meeting criterion or have a missing measurement at baseline and meeting criterion post-baseline.
m = Number of patients at risk (patients having a measurement not meeting the criterion or missing at baseline but at least one post-baseline measurement for the laboratory test(s) under consideration), n = Number of patients at risk who satisfy the criterion post-baseline.
QD: once daily, ALT: alanine aminotransferase, AST: aspartate aminotransferase, ALP: alkaline phosphatase, TBL: total bilirubin, ULN: upper limit of the normal range

**Table S4. Summary of patients with designated changes in QTcF and HR intervals (Safety set)**

| **Variable** | **Cipargamin 10 mg single dose N=10 n/m (%)** | **Cipargamin 10 mg**  **QD/3 days N=10 n/m (%)** | **Cipargamin 25 mg single dose N=12 n/m (%)** | **Cipargamin 25 mg**  **QD/3 days N=20 n/m (%)** | **Cipargamin 50 mg single dose N=21 n/m (%)** | **Cipargamin 50 mg  QD/3 days N=19 n/m (%)** | **Cipargamin 75 mg single dose N=21 n/m (%)** | **Cipargamin 150 mg single dose N=22 n/m (%)** | **Pooled  Artemether-lumefantrine N=51 n/m (%)** |
| --- | --- | --- | --- | --- | --- | --- | --- | --- | --- |
| **QTcF (ms)** |  |  |  |  |  |  |  |  |  |
| Increase > 30 ms to ≤ 60 ms | 0/9 | 1/10 (10.0) | 1/11 (9.1) | 5/20 (25.0) | 4/21 (19.0) | 7/19 (36.8) | 4/21 (19.0) | 2/22 (9.1) | 14/51 (27.5) |
| Increase > 60 ms | 0/9 | 0/10 | 0/11 | 2/20 (10.0) | 0/21 | 0/19 | 1/21 (4.8) | 0/22 | 0/51 |
| New > 450 ms and ≤ 480 ms | 0/9 | 0/10 | 1/12 (8.3) | 0/20 | 0/21 | 0/19 | 0/21 | 0/22 | 0/50 |
| New > 480 ms and ≤ 500 ms | 0/9 | 0/10 | 0/12 | 0/20 | 0/21 | 0/19 | 0/21 | 0/22 | 0/51 |
| New > 500 ms | 0/9 | 0/10 | 0/12 | 0/20 | 0/21 | 0/19 | 0/21 | 0/22 | 0/51 |
| **HR (bpm)** |  |  |  |  |  |  |  |  |  |
| Increase > 25% (HR > 100 bpm) | 0/9 | 0/10 | 0/11 | 0/20 | 0/21 | 0/19 | 0/21 | 2/22 (9.1) | 2/51 (3.9) |
| Decrease > 25% (HR < 50 bpm) | 0/9 | 0/10 | 0/11 | 1/20 (5.0) | 1/21 (4.8) | 0/19 | 1/21 (4.8) | 0/22 | 0/51 |

n: Number of patients who meet the designated criterion, m: Number of patients at risk for a designated criterion with at least one non-missing post-baseline value. For increase from baseline both baseline and a post-baseline value must be available. %=100*n/m, N: Total number of patients in the treatment group in this analysis set. HR: heart rate, bpm: beats per minute

**Cipargamin Case 1: 150 mg single dose, 2 CTCAE grade increase in ALT**

This subject, 32 years, female, experienced a grade 3 elevation in serum ALT on day 4, which was reported as serious. The baseline ALT level was already higher (grade 1), which started increasing from the next day of single dose of study drug and reached grade 3 level by day 4. The ALT values started reducing from day 5 and reached sub-baseline level at end of study. Considering positive temporal relationship and known safety profile, role of cipargamin in ALT elevation cannot be ruled out. Additionally, concomitant paracetamol may be a possible contributor. No related medical history was reported. The event was considered as related to study drug by investigator.

Table: Blood chemistry cipargamin case 1


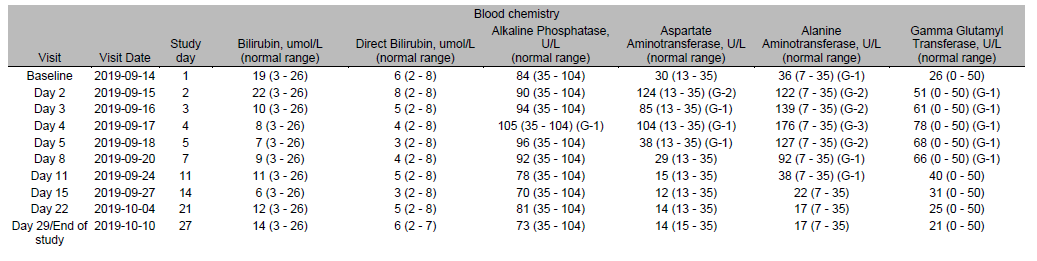


**Cipargamin Case 2: 10 mg single dose, 2 CTCAE grade increase in ALT**

This subject, 44 years, male, was treated with 10 mg single dose KAE609 and experienced an increase of 2 CTCAE grades in ALT on day 8. ALT levels normalized on day 29. Baseline value for ALT was within the normal range. The ALT increase started from day 3 onwards. No related medical history was reported and event was not seen as related to study drug by investigator. The patient received concomitant paracetamol from day 1 to day 3, which coincided with increase in ALT levels.

Table: Blood chemistry of cipargamin case 2


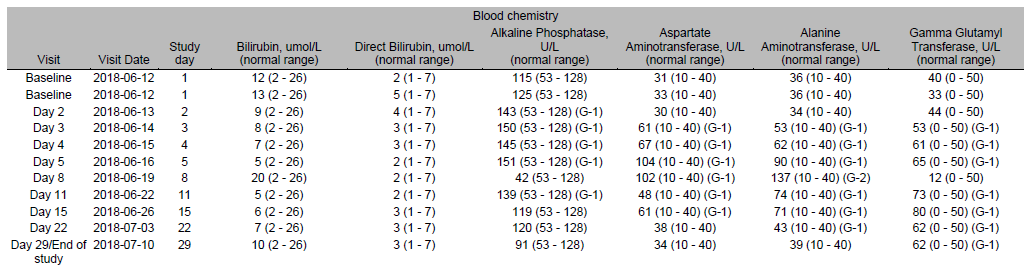


**Coartem Case 1:**

This subject, 34 years, male, from control arm (Coartem treatment) experienced an increase of 2 CTCAE grades in ALT on day 8. ALT levels normalized on day 22. Baseline value for ALT was within the normal range. No related medical history was reported. The patient received concomitant paracetamol.

Table: Blood chemistry for Coartem Case 1


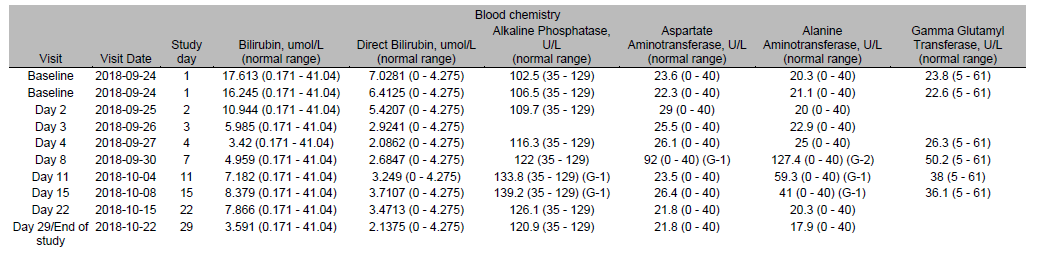


**Coartem Case 2: 2 CTCAE grade increase in AST on day 3**

This subject, 22 years, female, from control arm (Coartem treatment) experienced an increase of 2 CTCAE grades** in AST on day 3. AST levels normalized on day 8. Baseline value for AST was within the normal range. No related medical history was reported. The event was seen as related to study drug by investigator. Additionally, the patient received concomitant paracetamol from day 1 until day 4.

Table: Blood chemistry for Coartem Case 2


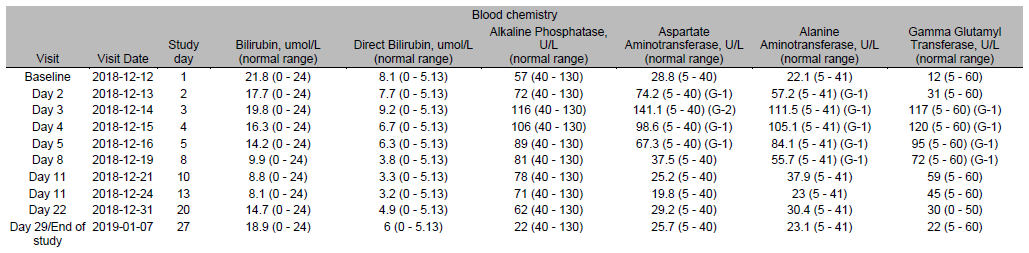

Supplement: Supplementary file 1 — Additional file 1. Supplement Hepatic safety and tolerability of cipargamin (KAE609). [file 12936_2021_4009_MOESM1_ESM.docx]
